# Efavirenz Dissolution Enhancement I: Co-Micronization

**DOI:** 10.3390/pharmaceutics5010001

**Published:** 2012-12-20

**Authors:** Maíra Assis da Costa, Rafael Cardoso Seiceira, Carlos Rangel Rodrigues, Cristiane Rodrigues Drago Hoffmeister, Lucio Mendes Cabral, Helvécio Vinícius Antunes Rocha

**Affiliations:** 1 Laboratory of Industrial Pharmaceutical Technology (LabTIF), Faculty of Pharmacy, Federal University of Rio de Janeiro, Rio de Janeiro, Brazil; E-Mails: mairacosta@far.fiocruz.br (M.A.C.); rangel@pharma.ufrj.br (C.R.R.); lmcabral@pharma.ufrj.br (L.M.C.); 2 Laboratory of Advanced Pharmaceutical Systems (LaSiFA), Farmanguinhos, FIOCRUZ, Rio de Janeiro, Brazil; E-Mail: cristianedrago@gmail.com; 3 Laboratory of Solid State Studies (LEES), Farmanguinhos, FIOCRUZ, Rio de Janeiro, Brazil; E-Mail: rcseiceira@far.fiocruz.br

**Keywords:** efavirenz, dissolution, micronization, poorly soluble drugs, sodium lauryl sulfate, polyvinylpyrrolidone

## Abstract

AIDS constitutes one of the most serious infectious diseases, representing a major public health priority. Efavirenz (EFV), one of the most widely used drugs for this pathology, belongs to the Class II of the Biopharmaceutics Classification System for drugs with very poor water solubility. To improve EFV’s dissolution profile, changes can be made to the physical properties of the drug that do not lead to any accompanying molecular modifications. Therefore, the study objective was to develop and characterize systems with efavirenz able to improve its dissolution, which were co-processed with sodium lauryl sulfate (SLS) and polyvinylpyrrolidone (PVP). The technique used was co-micronization. Three different drug:excipient ratios were tested for each of the two carriers. The drug dispersion dissolution results showed significant improvement for all the co-processed samples in comparison to non-processed material and corresponding physical mixtures. The dissolution profiles obtained for dispersion with co-micronized SLS samples proved superior to those of co-micronized PVP, with the proportion (1:0.25) proving the optimal mixture. The improvements may be explained by the hypothesis that formation of a hydrophilic layer on the surface of the micronized drug increases the wettability of the system formed, corroborated by characterization results indicating no loss of crystallinity and an absence of interaction at the molecular level.

## 1. Introduction

Although antiretroviral drug therapy has contributed significantly to improve patient quality of life and disease management, its use is associated with several drawbacks and inconveniences for patients. Associated severe side effects can be attributed to the high doses required to achieve a therapeutic effect, to inadequate drug concentration at the site of action, and/or to the poor bioavailability of some antiretroviral drugs. These drugs can present physico-chemical problems such as poor solubility that can lead to formulation difficulties [1].

**Figure 1:**
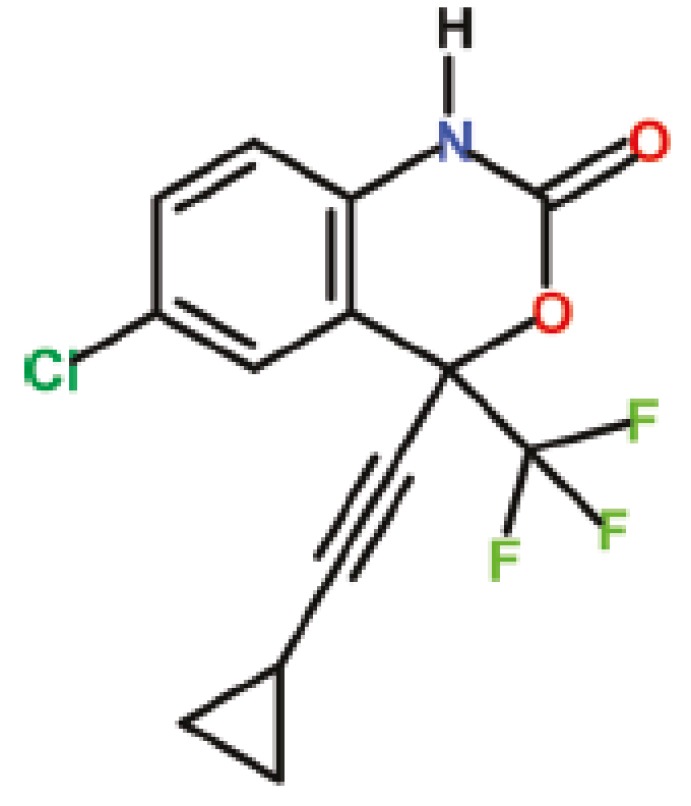
Chemical structure of Efavirenz [5].

Efavirenz (EFV) or (*S*)-6-chloro-4(cyclopropylethynyl)-1,4-dihydro-4-(trifluoromethyl)-2H-3, 1-benzoxazin-2-one, a non-nucleoside reverse transcriptase inhibitor (NNRTI) of the human immunodeficiency virus type 1 (HIV-1) [2,3], is a crystalline lipophilic solid with an aqueous solubility of 0.9 µg/mL and a low intrinsic dissolution rate of 0.037 mg/cm^2^/min [4]. The structure of EFV is relatively simple, although highly functionalized (Figure 1). Drugs whose intrinsic dissolution rate is less than 0.1 mg/cm^2^/min have dissolution as a rate-limiting step in absorption, pointing to the importance of dissolution improvement for EFV [4]. Moreover, EFV is categorized as Class II in the biopharmaceutics classification system (BCS), *i.e.*, it has low aqueous solubility and high membrane permeability, where alternative systems improving its solubility/dissolution are essential for satisfactory bioavailability.

Among the several methods available to achieve higher drug solubility or dissolution rates, galenical methods stand out as the most effective. Maximizing the porous structure of hydrosoluble polymeric matrix or incorporating superdisintegrants into formulations are basic approaches in pharmaceutical technology research to enhance the dissolution rate of poorly soluble drugs [6]. Physical modifications often aim to increase surface area, solubility and/or wettability of the powder particles and are therefore focused on size reduction or generation of amorphous states [7].

Micronization is a fast and relatively efficient process, which employs a fluid energy system mill that reduces particle size by impact and attrition using a high velocity stream of air. Micronization takes place immediately as a result of high-speed collisions among the particles suspended within the air stream [8]. At the micronizer, the materials suspended and transported at high velocity in a stream of air or steam pass through sprinklers at pressures of 100 to 150 pounds per square inch (psi). The violent turbulence of the air, or steam, reduces the particle size, mainly due to friction between particles but also with the walls of the equipment. Air is usually used because most pharmaceuticals have a low melting point or are thermolabile [9]. Many other systems are available to increase the dissolution of poorly soluble drugs, such as cyclodextrins [10,11], polymeric micelles [12,13,14,15,16,17], nanosuspensions [18,19,20] and lipidic formulations [21].

Of the few studies available in the literature focused on different formulations of EFV, most have not been dedicated to the concept of increased dissolution. Destache *et al.* [22] prepared poly (lactide-co-glycolide) (PLGA) nanoparticles containing ritonavir, lopinavir and efavirenz using water-in-oil-in-water homogenization, Yang and coworkers [23] prepared an amorphous dispersion containing EFV and polyvinylpyrrolidone (PVP) using spray-drying technology with methanolic solution forming solid solutions, and Madhavi and coworkers [4] prepared solid dispersions by solvent evaporation and physical mixture methods using polyethylene glycol (PEG) as the hydrophilic carrier. The results were not specific to EFV since other active ingredients were involved and the authors did not focuse on the dissolution profile obtained and performed an amorphous system that was inherently unstable, could recrystallize and had low physical stability

For all these studies, the level of evidence for benefits in terms of dissolution improvement is not clearly described. Moreover, among the studies available, none report complete information regarding design, characterization and biological evaluation of the formulations assessed. The industrial feasibility for the proposed systems is also questionable. Some papers have been published focusing on polymorphic changes in active ingredients, but there is no clear evidence of dissolution improvement.

The development of coprocessed drugs is an area of great interest in pharmaceutical technology due to easy processability and the possibility of enhancements in bioavailability. Efavirenz delivery using this kind of system is a good strategy for AIDS treatment, because increasing bioavailability can reduce the dose needed for therapeutic efficacy.

Efavirenz API is now commercially available as micronized powder. Micronization is an established manufacturing process and has been mastered from a technological point of view. Moreover, it has other key advantages: The process is dry and scale-up is relatively straightforward. The micronization process is currently being used commercially to provide the pharmaceutical industry with efavirenz in micronized powder form. The process is well understood technologically and offers the advantage of being dry and feasible on an industrial scale. Jain and coworkers [24] showed the effectiveness of co-micronization for decreasing particle size of poorly water-soluble drugs. The study also compared the impact of co-micronization *versus* micronization of pure drug. These authors also reported that micronization of the poorly soluble drug alone could generate hydrophobic poorly wettable surfaces. The excipients used in co-micronization adhere to the drug surface, thereby facilitating wetting and dissolution. The study, along with the others cited above, provided the underlying basis for the present work on co-micronization.

Against this background, the aim of the present work was to develop and characterize co-processed systems containing efavirenz and sodium lauryl sulfate (SLS) or polyvinylpyrrolidone (PVP) for dissolution improvement using the micronization process. 

## 2. Results and Discussion

### 2.1. Scanning Electron Microscopy (SEM)

**Figure 2:**
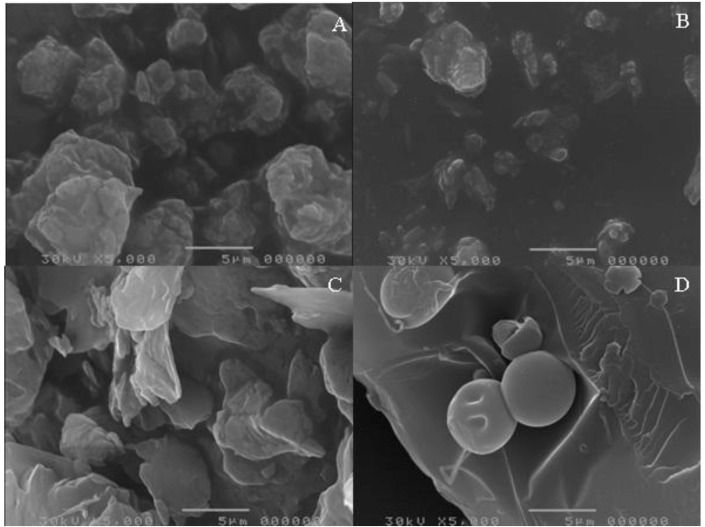
(**A**) Photomicrographs of unprocessed efavirenz (EFV); (**B**) micronized EFV; (**C**) unprocessed sodium lauryl sulfate (SLS); and (**D**)unprocessed polyvinylpyrrolidone (PVP).

**Figure 3:**
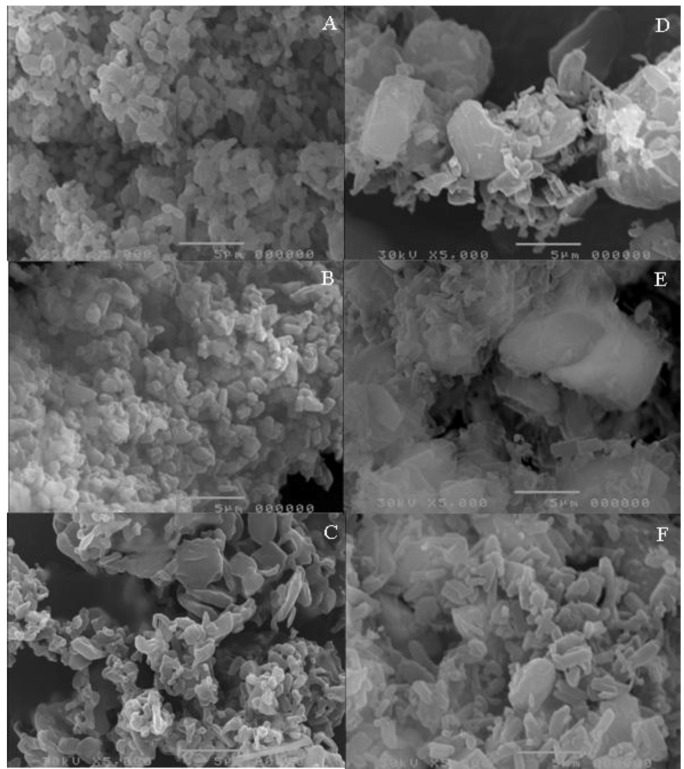
Photomicrographs of co-micronized mixtures EFV:SLS (**A**) (1:0.25); (**B**) (1:0.50) and (**C**) (1:1) and (**D**)EFV:PVP (1:0.25); (**E**) (1:0.50) and (**F**) (1:1).

Figure 2 shows the photomicrographs of unprocessed and micronized EFV, unprocessed SLS and unprocessed PVP. A difference in morphology of the two dispersants is evident, as is the lower particle size of the micronized drug. Using direct measurement, the particle size of unprocessed and processed drug was 5.9 µm and 3.0 µm, respectively, showing a significant size decrease after processing. However, in practical terms, this size decrease may not be relevant for the processing or the dissolution of the material. The particle size of SLS and unprocessed PVP was 5.0 µm and 36.3 µm, respectively. Figure 3 depicts the photomicrographs of co-micronized mixtures.

In general, distinct regions for drugs and carrier are not visible on the coprocessed photomicrographs, thus presenting a homogeneous system. Visualization could be achieved by spectroscopic techniques, such as Raman or Fourier transform infrared spectroscopy (FTIR) microscopy, but this detection is beyond the scope of the present paper.

The images of the co-micronized systems (Figure 3) reveal that co-processed EFV:SLS particles are more homogeneous in size and morphology than co-processed EFV:PVP, and also have a smaller particle size.

Taking into account drug:excipient proportions, the lower the amount of carrier in the mix, the higher its particle size. The particle size range among EFV:SLS mixtures was smaller than among EFV:PVP, a finding which may be due to the larger average particle size of unprocessed PVP compared to unprocessed SLS. The values for average particle size of the co-micronized mixtures, measured directly, are listed in Table 1.

**Table 1:** Average particle size, measured directly.

	Particle size (µm)	
	SLS	PVP
EFV:dispersant (1:0.25)	1.9 µm	6.1 µm
EFV:dispersant (1:0.5)	1.7 µm	4.7 µm
EFV:dispersant (1:1)	1.5 µm	2.5 µm

### 2.2.Fourier Transform Infrared (FTIR) Spectroscopy

The samples were analyzed in the 2500–500 cm^−1^ range, within which the most important peaks for the evaluation of efavirenz lie.

FTIR spectrum of unprocessed EFV showed characteristic bands, similar to the spectrum obtained by Shown *et al.* [25]. The characteristic infrared absorption bands of EFV are listed in Table 2.

The same bands found in the unprocessed EFV spectrum are also present in the micronized EFV spectrum. This remained unchanged after micronization; therefore processing did not interfere with the structure of the drug at the molecular and crystalline level. Similarly, the excipient spectra showed no modification after micronization.

**Figure 4:**
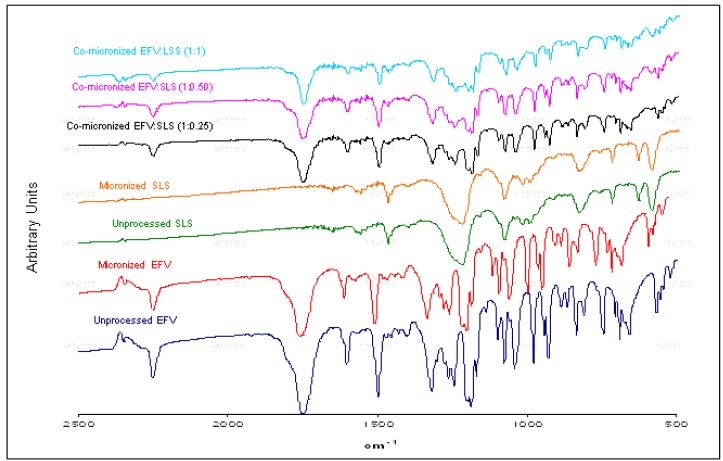
FITR spectra of unprocessed and micronized efavirenz and SLS and co-micronized mixtures of EFV:SLS (1:0.25), (1:0.50) and (1:1).

The EFV:SLS co-micronized system spectra proved similar to the corresponding physical mixture spectra (Figure 4), indicating no molecular change after micronization for all ratios tested.

**Table 2:** Characteristic infrared absorption bands of EFV.

Frequency (cm^−1^)	Vibrational assignments
2260	Typical exocyclic triple bond stretching
1757	C=O stretching
1602	Tertiary amide
900–650	Aromatic ring
1350–1120	CF_3_
1096–1089	C-Cl stretching

Characteristic bands can be observed in all samples analyzed. Comparing the spectra of the three proportions tested again revealed no significant differences. A slight difference in band intensity was evident, but was related to the component concentrations in the mixture.

The characteristic bands of each individual component of the mixture were evident, indicating no molecular interaction.

Akin to EFV:SLS, the co-micronized systems of EFV:PVP also presented bands in the same regions as the corresponding physical mixture, although PVP has a carbonyl group, which has often been reported as a hydrogen bond acceptor [26], while EFV has the possibility of hydrogen bond formation by the presence of the N–H group.

**Figure 5:**
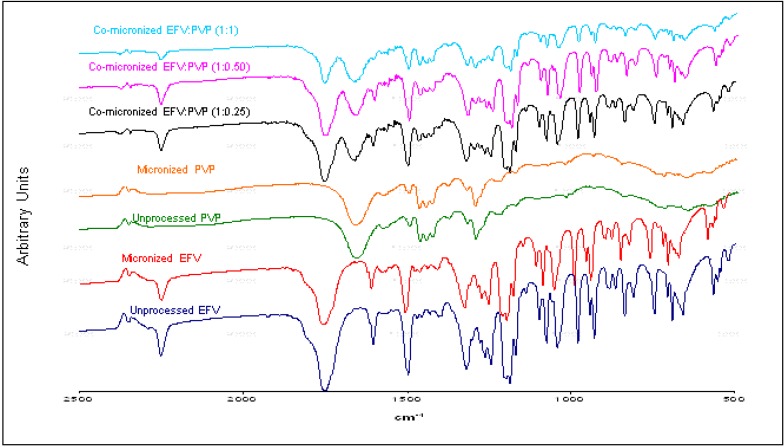
FITR spectra of unprocessed and micronized efavirenz and PVP and co-micronized mixtures EFV:PVP (1:0.25), (1:0.50) and (1:1).

The spectra of the three proportions of EFV:PVP tested in co-micronization are shown in Figure 5, comparing unprocessed and micronized EFV and PVP. Similarly to co-micronized mixtures of EFV:SLS, only slight differences in band intensity are evident. Earlier studies on micronization obtained similar results [27,28]. 

### 2.3. Differential Scanning Calorimetry (DSC)

**Figure 6:**
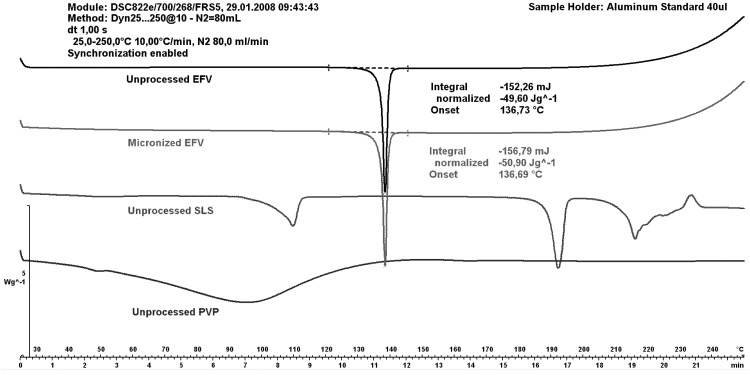
Differential scanning calorimetry (DSC) curves of unprocessed efavirenz, micronized efavirenz, unprocessed SLS and unprocessed PVP.

Figure 6 shows differential scanning calorimetry (DSC) curves of unprocessed excipients as well as both unprocessed and micronized efavirenz. The efavirenz endothermic peak was observed at 137 °C, similar to the peak value reported in the literature [4]. These authors attributed the temperature range of 135.27–139.79 °C to the melting of efavirenz.

The peak for the micronized drug was similar to that of the unprocessed drug; indicating maintenance of crystallinity after micronization without hydrophilic carriers. The SLS curve depicts a first event; probably due to water loss; and a second attributed to melting point. The PVP showed no events given its amorphous nature.

The carriers micronized separately were analyzed (data not shown), demonstrating that size reduction of SLS resulted in faster water loss (the corresponding peak was displaced to a lower temperature). Micronization for PVP resulted in slower water loss.

**Figure 7:**
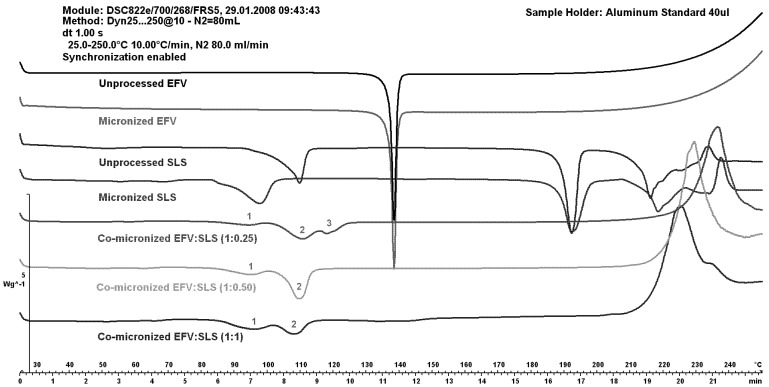
DSC curves of unprocessed and micronized efavirenz and SLS, compared to mixtures co-micronized at the proportions (1:0.25), (1:0.50) and (1:1).

Figure 7 shows DSC curves of EFV:SLS co-micronized mixtures (1:0.25), (1:0.50) and (1:1) compared to both unprocessed and micronized EFV and SLS.

The peaks of SLS (1 and 2) increased with increasing SLS concentration in the co-micronized mixture. The peak of the proportion (1:0.50) was higher than for the proportion (1:1), probably due to homogeneity. The peak corresponding to the melting point of EFV (3) was higher with increased amount of drug in the mixture. These results may indicate interaction between components. The profile of physical mixtures was similar to that of co-processed materials, indicating no influence of the process. 

Using PVP as the carrier for micronization, the peak for EFV melting almost disappeared at all proportions tested. On DSC curves of the physical mixture (data not shown), in contrast to that shown in co-micronized mixtures, the peak decreased with decreasing drug concentration in the mixture, and water loss of PVP was observed. 

**Figure 8:**
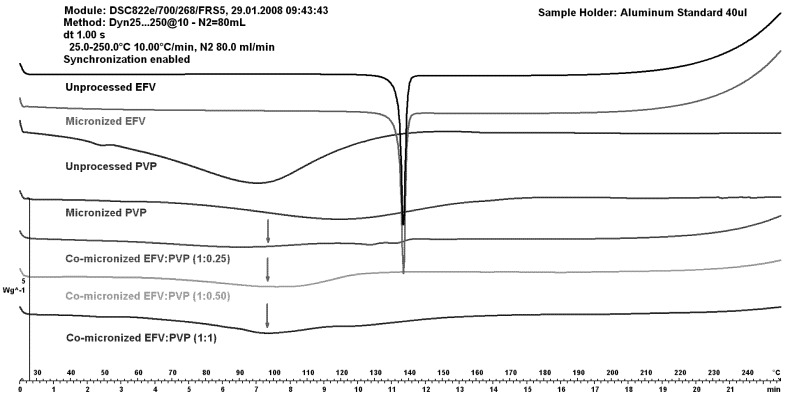
DSC curves of unprocessed and micronized efavirenz and PVP, compared to the co-micronized mixture proportions of (1:0.25), (1:0.50) and (1:1).

The DSC curves of unprocessed and micronized EFV and PVP are shown in comparison to co-micronized mixtures at (1:0.25), (1:0.50) and (1:1) in Figure 8.

An increase in the peak of PVP can be observed (with increasing PVP concentration), and disappearance of the drug peak, which could indicate some interaction between components caused by processing. This was significantly different to the result obtained with SLS as the carrier.

Comparing DSC curves of EFV:SLS and EFV:PVP in different proportions, it could be inferred that there was an interaction between the drug and the carriers during heating, based on the changes seen in the peaks compared to the unprocessed drug. This possible interaction can occur from temperatures of around 80 °C. Interaction may occur in the form of partial amorphization, in other words, part of the drug loses crystallinity under analysis conditions (this can be refuted or confirmed by X-ray diffraction) or through the carrier interacting with the drug, possibly solubilizing EFV with analysis heating or as the system suffers degradation. 

### 2.4. Thermogravimetric Analysis (TGA)

**Figure 9:**
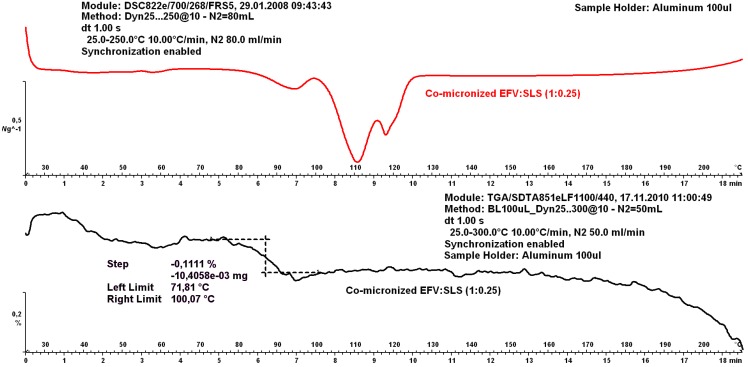
Thermogravimetric analysis (TGA) curves of co-micronized mixture EFV:SLS proportion (1:0.25) compared to DSC curve.

Only the sample EFV:SLS (1:0.25) was analyzed to confirm water loss from SLS given the characteristic peak evident in DSC analysis. The thermogravimetric analysis (TGA) curve is represented in Figure 9.

It is clear that the first endothermic peak in DSC corresponds to the weight loss noted in the TGA curve. This means that this peak is related to water loss, having no relation to material crystallinity. The amount of water incorporated was tiny in both samples. The following two endothermic peaks are therefore related to structural transitions of the material, indicating a possible interaction between EFV and excipients, as previously discussed. 

### 2.5. Hot-Stage Microscopy (HSM)

**Figure 10:**
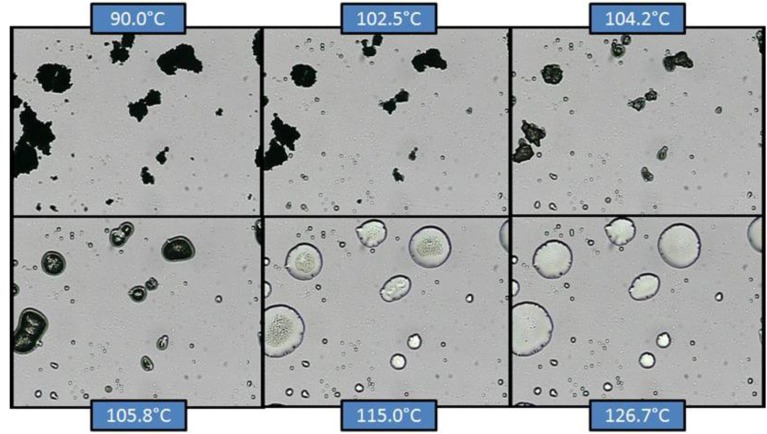
Hot-stage microscopy (HSM) of co-micronized EFV:SLS (1:0.25).

**Figure 11:**
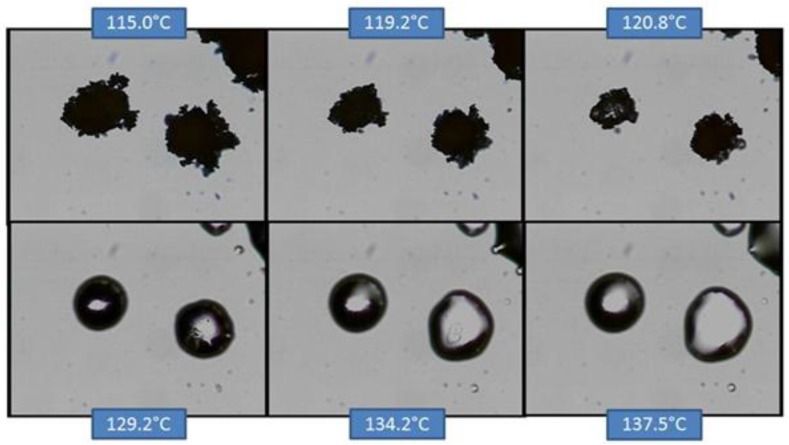
HSM of co-micronized EFV:PVP (1:0.25).

Figure 10 and Figure 11 show the results from co-micronized samples (1:0.25) of EFV:SLS and EFV:PVP, respectively.

In co-micronized samples with SLS, it was not possible to observe the water loss detected by TGA, since the percentage of solvent was too small. However, it was possible to detect the SLS melting point and the formation of a solution with EFV microcrystals. With continuous heating, EFV melting was evident. The temperatures seen are consistent with the transitions observed in the DSC analysis. The profiles were similar for the three proportions tested. The sequence of transitions confirms the hypothesis previously suggested by DSC.

In the case of co-micronized samples with PVP, the three profiles were very similar, and it was not possible to identify any influence of PVP concentration. Unlike the situation with SLS, EFV melting can be seen concomitantly with PVP solubilization, and this dissolves the EFV crystals before they melt. This profile confirms those observed in the DSC curves, where it was not possible to observe the peak corresponding to EFV melting.

Thus, hot-stage microscopy proved to be a useful tool for the elucidation of DSC results, allowing visual observation of thermal behavior of the samples. This analytical tool has been used in the study and characterization of crystalline drugs, but its use for the evaluation of co-micronized systems was not found in the literature. In this study, the technique contributed significantly to help compare samples, confirming the hypothesis drawn from the evaluation of data derived from DSC analysis. 

### 2.6. Powder X-ray Diffraction (DRX)

**Figure 12:**
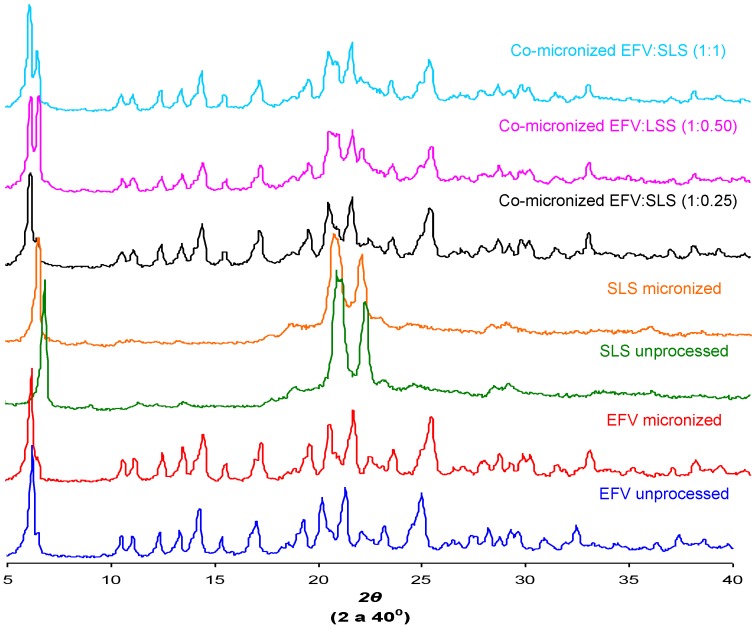
X-ray diffraction patterns of unprocessed and micronized EFV and SLS, compared to co-micronized EFV:SLS mixtures at the proportions (1:0.25), (1:0.50) and (1:1).

The crystal structure of a substance is an important characteristic that can influence solubility [29]. The X-ray diffraction patterns found for unprocessed EFV and excipients, processed excipients alone and co-processed systems are depicted in Figure 12.

Peak intensity is affected by crystal size and crystallinity [30]. The peak intensity of the diffraction patterns of micronized and co-processed drug would therefore be expected to be lower than the pure drug or present a displacement peak due to decreasing particle size and/or amorphization.

The diffraction patterns obtained for the unprocessed drug were similar to those reported in the literature [5]. The same peaks were found in diffraction patterns of micronized EFV and all co-processed samples, identifying the principal diffraction angles (2θ) as 6.20°, 20.20°, 21.35° and 25.00°.

**Figure 13:**
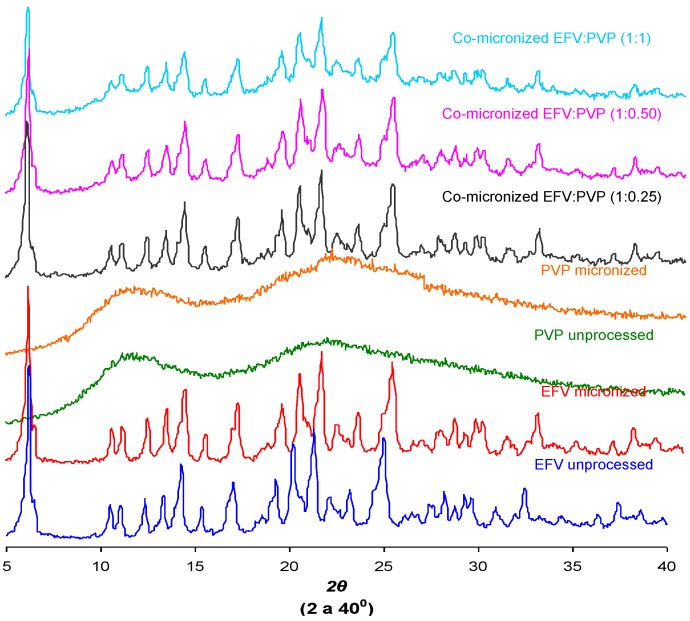
X-ray diffraction patterns of unprocessed and micronized EFV and PVP, compared to co-micronized mixtures of EFV:PVP in the proportions (1:0.25), (1:0.50) and (1:1).

Figure 12 and Figure 13 show the X-ray diffraction patterns obtained for co-micronized mixtures with SLS and PVP, in comparison with the unprocessed and micronized drug and the carrier. The mean peaks found in unprocessed EFV can also be seen in the diffraction patterns of the co-processed mixtures. In conjunction with these peaks, the main peaks obtained for SLS, 6.85°, 20.55° and 21.90° are evident, while PVP shows no peak on the X-ray diffraction analyses because of its amorphous nature.

 Only the characteristic peaks of each component could be identified in diffraction patterns of co-processed mixtures, and there were no significant changes in peak intensity and/or position compared to those of unprocessed EFV and excipients for all proportions tested. This result indicates the maintenance of the crystal habit of the substances, which does not confirm the amorphization disclosed by thermal analysis results. Thus, this phenomenon may have occurred due to heating during DSC analysis, as amorphization does not occur at room temperature and/or as a result of the process. The characteristic peaks of drug and carriers in the diffraction patterns of the co-processed mixtures also indicates that there was no degradation of the mixture as a result of processing, since it occurs when the peaks are not evident in the analysis. Thus, the possibility of degradation due to reduction and disappearance of peaks in DSC results was also not confirmed. 

Previous results obtained in studies with fenofibrate [7], glybuzole [31], carbamazepine [27] and EMD 57033 [32], that used micronization, also showed that maintenance of a crystal structure after processing, akin to the EFV in the present study.

The co-micronization process is rarely described in the literature. The dissolution improvement of poorly water-soluble drugs for co-milling with surfactants, such as SLS, was also investigated [31], however, an amorphous state was generated. Although conversion to the amorphous state can significantly improve solubility and dissolution, this state can revert to a lower energy condition, normally crystalline forms, during storage. Unfortunately, conversion time is not easy to predict. A formulation that offers a drug with rapid dissolution, but with crystalline API form therefore represents an ideal solid dosage form for oral administration [32].

This paper failed to observe amorphization of the drug, although an increase in dissolution was seen, as verified by Jagadish and coworkers [33], whose study showed increased dissolution and bioavailability without amorphization, simply by enhancing the wettability of the particles. 

### 2.7. Powder Dissolution Studies

The co-micronized mixtures presented enhanced dissolution profiles in comparison with those obtained for unprocessed EFV, micronized EFV and physical mixtures, for all proportions assessed. According to the results of difference (*f*1) and similarity (*f*2) factors, the dissolution profile of co-micronized mixtures can be deemed significantly different to the unprocessed drug profile for all proportions tested (Table 3). The co-micronized EFV:SLS mixtures also showed significant differences compared to physical mixtures (data not shown). This statement is based on the classification actually considered by regulatory agencies such as Food and Drug Administration (FDA) and European Medicines Agency (EMA).

**Table 3:** Values of *f*1 and *f*2 found for processed samples compared to the unprocessed drug.

Processed	EFV unprocessed	
	*f*1	*f*2
EFV micronized	16.45	51.98
EFV:SLS (1:0.10)	68.56	21.19
EFV:SLS (1:0.25)	93.45	14.25
EFV:SLS (1:0.50)	98.75	13.07
EFV:SLS (1:1)	104.56	11.87
EFV:PVP (1:0.25)	60.81	23.68
EFV:PVP (1:0.50)	56.48	24.64
EFV: PVP (1:1)	49.17	26.94

**Figure 14:**
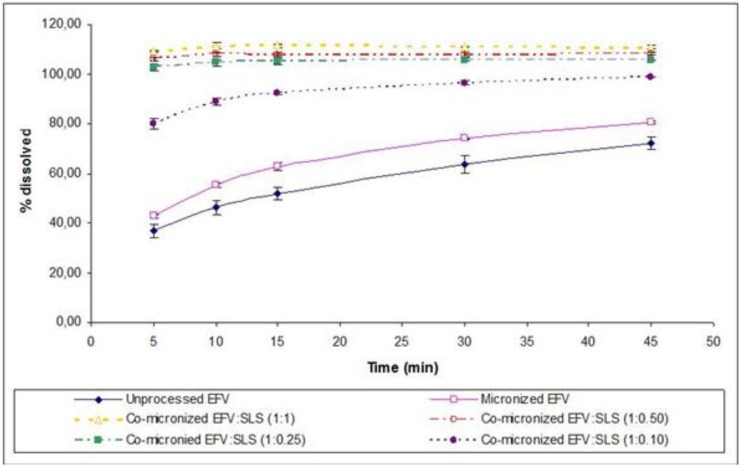
Powder dissolution profile of EFV in SLS 0.5% (*n*=3) compared to micronized and unprocessed EFV and co-micronized EFV:SLS mixtures at the proportions (1:0.10), (1:0.25), (1:0.50) and (1:1).

According to the same calculation, the three proposed major proportions showed powder dissolution profiles similar to each other (Table 4), where proportions of EFV:SLS no higher than (1:0.25) were required for full and fast dissolution of EFV. These powder dissolution profiles are shown in Figure 14.

**Table 4:** Values of* f*1 and *f*2 found for EFV:SLS processed samples.

Processed	EFV:SLS (1:0.25)	
	*f*1	*f*2
EFV:SLS (1:0.50)	2.74	75.49
EFV:SLS (1:1)	5.74	60.59

The EFV:PVP co-micronized, as well as EFV:SLS, mixtures showed dissolution profiles which were significantly different to those of the unprocessed drug (Table 3) and the physical mixtures (data not shown) for all proportions. It is noteworthy that, in the initial points, more rapid dissolution was observed while final values did not show such a significant increase, contrary to that shown in the case of co-micronized EFV:SLS samples.

**Figure 15:**
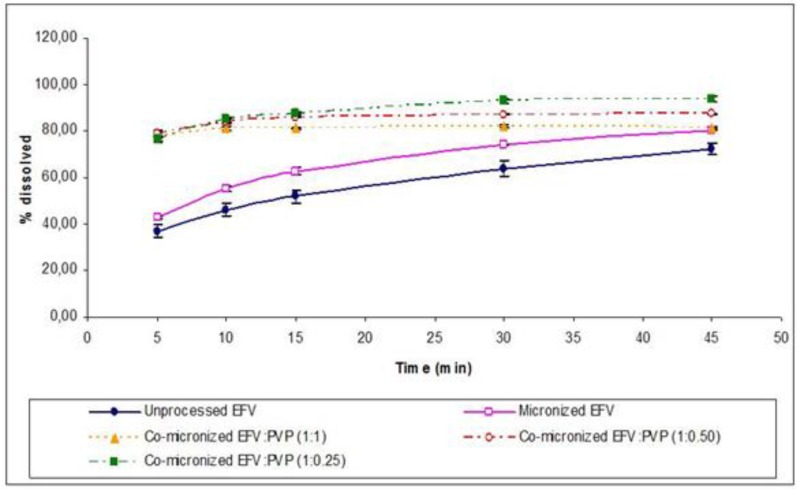
Powder dissolution profile of EFV in SLS 0.5% (*n*=3) compared to micronized and unprocessed EFV and co-micronized EFV:PVP mixtures at the proportions (1:0.25), (1:0.50) and (1:1).

Figure 15 shows the powder dissolution profile of co-micronized mixtures compared with unprocessed and micronized EFV. The three proportions tested all had similar powder dissolution profiles (Table 5).

**Table 5:** Values of* f*1 and *f*2 found for EFV:PVP processed samples.

Processed	EFV:PVP (1:0.25)	
	*f*1	*f*2
EFV:PVP (1:0.50)	3.99	68.48
EFV:PVP (1:1)	7.84	54.83

The size reduction method has been extensively used because the increase in surface area can enhance dissolution rate, and consequently, the bioavailability of pharmaceutical materials [27]. Nevertheless, it cannot be asserted that the enhancement obtained in the dissolution profile of the co-micronization mixtures, both with SLS and PVP, was due solely to particle size decrease, in spite of the scanning electronic microscopy analyses showing alterations in particle size after co-processing (Figure 3). As in the case of results found by Vogt and collaborators [32], it can be hypothesized that a formed hydrophilic layer surrounds the drug, enhancing wettability and leading to more rapid dissolution of the co-micronized mixture.

The results of powder dissolution of co-micronized mixtures with PVP showed lower values compared to co-micronized mixtures using SLS. All profiles obtained for co-micronized mixtures with SLS differed from those of co-micronized EFV:PVP (Table 6). Generally, polymers are known to be able to surround fine drug crystals, hindering their recrystallization from solution by reducing the surface area for crystallization on the drug particles, but this effect can also hinder dissolution by forming a barrier against penetration of water molecules [7]. The profiles obtained for co-micronized EFV:PVP mixtures, predominantly at 45 min, evidenced a reduction in dissolution with increasing PVP concentration. Using SEM, greater homogeneity of particles of co-micronized mixtures with SLS was seen in comparison to co-micronized mixtures with PVP (Figure 3), which can consequently influence the wettability and dissolution of systems.

**Table 6:** Values of* f*1 and *f*2 found for EFV:PVP processed samples compared to EFV:SLS proportions.

**Processed**	**EFV:SLS (1:0.25)**	
	***f*1**	***f*2**
EFV:PVP (1:0.25)	16.88	36.71
EFV:PVP (1:0.50)	19.11	34.81
EFV:PVP (1:1)	22.89	30.98
**Processed**	**EFV:SLS (1:0.50)**	
	***f*1**	***f*2**
EFV:PVP (1:0.25)	19.09	33.54
EFV:PVP (1:0.50)	21.27	31.89
EFV:PVP (1:1)	24.95	28.53
**Processed**	**EFV:SLS (1:1)**	
	***f*1**	***f*2**
EFV:PVP (1:0.25)	21.39	30.62
EFV:PVP (1:0.50)	23.51	29.11
EFV:PVP (1:1)	27.08	26.13

In order to achieve the lowest possible proportion of carrier in the formulation, on the basis of the excellent results obtained in powder dissolution of the co-micronized mixture, particularly with SLS, a smaller proportion was tested: EFV:SLS (1:0.10). The dissolution profile is depicted in Figure 14 compared to the unprocessed and micronized drug and all the proportions of co-micronized mixtures with SLS previously tested. The new dissolution profile obtained was lower than the other proportions tested and was considered to be significantly different according to the *f*1 and *f*2 factors, in spite of being higher than in the unprocessed and micronized drug (Table 7). The small quantity of carrier may have hindered the formation of the hydrophilic layer at the drug surface. Nevertheless, even a small quantity of carrier co-micronized with EFV was able to provide significant improvement in dissolution, emphasizing the importance of co-micronization. In addition, the results of dissolution of the co-micronized mixtures were higher than those obtained for the physical mixture, thus confirming the merit of the process studied.

**Table 7:** Values of* f*1 and *f*2 found for processed EFV:SLS (1:0.10) compared to the unprocessed and micronized drug and other EFV:SLS proportions.

**Processed**	**EFV:SLS (1:0.10)**	
	***f*1**	***f*2**
EFV unprocessed	40.68	21.19
EFV micronized	30.92	26.82
EFV:SLS (1:0.25)	14.77	41.85
EFV:SLS (1:0.50)	17.91	37.97
EFV:SLS (1:1)	21.35	34.49

Based on these results, co-micronization appears to be an efficient technique for EFV processing to enhance the dissolution profile. It can lead to greater drug bioavailability, as Vogt and colleagues [7] demonstrated for fenofibrate. According to Mooter *et al.* [34], a lack of crystallinity, increased wettability, and reduced drug particle size, were considered to be predominant factors in controlling dissolution. Barzegar-Jalali and coworkers [28] proposed that, in addition to these factors, the deaggregation promoted by carriers is the underlying reason for enhanced drug dissolution.

Events related to drug amorphization were not responsible for the improvement in dissolution rate obtained, since the powder X-ray diffraction (DRX) results confirmed crystallinity maintenance. The possibility of interaction at molecular and structural levels was also ruled out given the similarity of infrared spectra obtained from co-processed and physical mixtures for all proportions tested, showing neither band displacement nor enlargement. Particle size reduction was observed in some cases by SEM, which may have influenced the increased dissolution rate of some co-processed samples, especially the co-micronized mixtures containing SLS. This sample attained the highest dissolution results, and presented the lowest particle size. The enhanced wettability, the solubilizing effect of the carrier, the drug dissolution in hydrophilic support and/or a combination of these factors, represent possible hypotheses to explain the enhancement of the powder dissolution profile obtained by co-micronization, but further testing is needed to confirm this theory.

## 3. Experimental Section

### 3.1. Co-Processed Preparation

The drug (efavirenz produced by a Brazilian chemistry company whose name will not be disclosed due to a confidentiality agreement) and the excipients PVP K30 (Jiaozuo Meida Fine Chemicals, Shangai, China) and SLS (Vetec, Rio de Janeiro, Brazil), were manually mixed in the appropriate ratios and the resultant mixture was then micronized in a Ultra Jet model 50 micronizer (Zelus, São Paulo, Brazil). The drug and excipients were micronized separately for comparison. EFV:SLS ratios were (1:1), (1:0.50), (1:0.25) and (1:0.10), while EFV:PVP ratios were (1:1), (1:0.50) and (1:0.25). A process pressure of 4.0 kgf/cm^2^ and atomization pressure of 6.0 kgf/cm^2^ were used during micronization.

### 3.2. Scanning Electron Microscopy (SEM)

The average particle size, size distribution and morphology were examined using an EM 906 SEM (Carl Zeiss, Oberkochen, Germany). The samples were mounted on an aluminum stage using adhesive carbon tape and coated with gold under an argon atmosphere in a high vacuum evaporator. 

### 3.3. Fourier Transformed Infrared (FTIR) Spectral Studies

Infrared spectroscopy is a method of analysis that provides information about the functional groups present in the molecular structure of substances. The FTIR analyses were done to confirm the occurrence of structural changes at a molecular level as a result of co-processing of EFV with the carriers SLS and PVP.

The FTIR spectrum was recorded in transmission mode on a Prestige FTIR 8000 spectrometer (Shimadzu, Kyoto, Japan). The analyses were conducted applying spectroscopy Fourier transformed infrared (FTIR) where band positions are presented in wavenumbers (v) usually expressed in inverse centimeters (cm^−1^), and band intensities expressed as transmittance (T). Approximately 3 mg of each sample was weighed and mixed with potassium bromide then compressed in a hydraulic press under 10 T of pressure for 1 min.

### 3.4. Differential Scanning Calorimetrty (DSC)

This technique was used to evaluate possible crystalline changes or drug degradation. DSC curves were collected using a calorimeter model 822 (Mettler Toledo, Ohio, USA). Samples were analyzed at a temperate range of 25 °C to 250 °C using a heating rate of 10 °C/min. The samples were weighed in open aluminum pans. An empty pan was used as a reference.

### 3.5. Thermogravimetric Analysis (TGA)

A thermogravimetric analyzer model 851 (Mettler Toledo, Ohio, USA) was used. Approximately 10 mg of each sample was weighed in aluminum pans. Sample mass was monitored and the temperature was increased from 25 °C to 300 °C at a rate of 10 °C/min.

### 3.6. Hot-Stage Microscopy (HSM)

Hot-stage microscopy is a thermoanalytical technique in which the optical property of the sample is monitored against temperature or time, while the temperature of the sample, under a specified atmosphere, is programmed. The information collected during visual analysis is valuable for the confirmation of physical changes detected in the DSC analysis. The heating rate should be the same as that used in the DSC analysis, allowing direct comparison between results. HSM is required to confirm transitions such as melts and recrystallizations.

Hot-stage microscopy was conducted using a FP 82 heating cell and an SP 90 temperature controller (both by Mettler Toledo, Ohio, USA) with an optical light microscope BX 50 (Olympus, Tokyo, Japan). Images were obtained at a heating rate of 10 °C/min, in a temperature range of 30 °C to 200 °C.

### 3.7. Powder X-ray Diffraction (PXRD)

The measurements were carried out using an X-ray diffractometer (Rigaku, Tokyo, Japan) and the operating conditions were as follows: Cuk_α_ radiation, voltage 30 kv, current 15 mA and time constant 0.05°/s. The wavelength used was λ = 1.5418 Å.

### 3.8. Powder Dissolution Studies

The powder dissolution method has been reported in the literature [35,36]. The tests were carried out using a bathless dissolution system Evolution 6100 from Distek (New Jersey, USA). Powder samples containing 600 mg of efavirenz (dosage presented in reference drug product) were placed into dissolution vessels and stirred at 50.0 ± 0.1 rpm using the paddle method (USP [37]-apparatus II). The media used was 900 mL of aqueous solution with 0.5% of sodium lauryl sulfate (method developed in-house). The temperature was maintained at 37.0 ± 0.2 °C. The dissolved solution samples of 10 mL were collected at 5, 10, 15, 30 and 45 min and filtered through a 0.45 µm pore membrane. Samples were analyzed using an UV spectrophotometer (Shimadzu, Kyoto, Japan) and absorbance measured at 248 nm. The dissolution test was performed three times for each sample.

The powder dissolution profiles were compared using a model-independent method, based on calculation of difference (*f*1) and similarity (*f*2) factors (Microsoft Excel). Two dissolution profiles were considered to be similar when *f*1 had values between 0 and 15 and *f*2 had results between 50 and 100 [38].

## 4. Conclusions

The proposed study clearly presents a technological challenge, namely, enhancement of EFV dissolution. To achieve this objective, the formation of agglomerates via co-micronization was proposed, using SLS and PVP as dispersant agents. Dissolution enhancement of EFV was evident for both carriers and all the proportions tested showed higher powder dissolution profiles than the unprocessed drug. The process was not able to change the crystallinity pattern of the drug, achieving significant improvement of the dissolution profile of EFV, without the amorphization of the API. This result allows the conclusion that there is no concern about any transformation from amorphous to crystalline structure, which could result in a decrease of dissolution during stability. The SLS proved to be the best hydrophilic carrier in co-micronization in comparison to PVP and the proportion EFV:SLS (1:0.25) was superior than the others tried, while also being more suitable for tableting, considering that less powder will be present in the formulation. The results of the present work proved to be very promising in terms of industrial applications, but further tests are needed for full characterization of the formulated material and scale-up studies should be performed.

## References

[1] Ojewole E., Mackraj I., Naidoo P., Govender T. (2008). Exploring the use of novel drug delivery systems for antiretroviral drugs. Eur. J. Pharm. Biopharm..

[2] Mishra S., Chaturvedi D., Srivastava A., Tandon P., Ayala A.P., Siesler H.W. (2010). Quantum chemical and experimental studies on the structure and vibrational spectra of efavirenz. Vib Spectrosc..

[3] Ribeiro J.A.D., Moreira de Campos L.M., Alves R.J., Lages G.P., Pianetti G.A. (2007). Efavirenz related compounds preparation by hydrolysis procedure: Setting reference standards for chromatographic purity analysis. J. Pharm. Biomed..

[4] Madhavi B.B., Kusum B., Krishna Chatanya C.H., Madhu M.N., Sri Harsha V., Banji D. (2011). Dissolution enhancement of efavirenz by solid dispersion and PEGylation techniques. Int. J. Pharm. Investig..

[5] Mahapatra S., Thakur T.S., Joseph S., Varughese S., Desiraju G.R. (2010). New solid state forms of the anti-HIV drug efavirenz. Conformational flexibility and high *Z*’ issues. Cryst. Growth Des..

[6] Rudnic E.M., Schwartz J.B., David B.T. (2000). Oral solid dosage forms. Remington: The Science and Practice of Pharmacy.

[7] Vogt M., Kunath K., Dressman J.B. (2008). Dissolution enhancement of fenofibrate by micronization, cogrinding and spray-drying: Comparison with commercial preparations. Eur. J. Pharm. Biopharm..

[8] Omelczuk M.O., Wang C.C., Pope G. (1996). Influence of micronization on the compaction properties of an investigational drug using tableting index analysis. Eur. J. Pharm. Biopharm..

[9] Kanig J., Lachman L., Lieberman H. (2001). Teoria e Prática na Indústria Farmacêutica.

[10] Makhlof A., Miyazaki Y., Tozuka Y., Takeuchi H. (2008). Cyclodextrins as stabilizers for the preparation of drug nanocrystals by the emulsion solvent diffusion method. Int. J. Pharm..

[11] Miyamoto Y., Nakahara M., Motoyama K., Ishiguro T., Oda Y., Yamanoi T., Okamoto I., Yagi A., Nishimura H., Hirayama F. (2011). Improvement of some physicochemical properties of arundic acid, (*R*)-(−)-2-propyloctanonic acid, by complexation with hydrophilic cyclodextrins. Int. J. Pharm..

[12] Chiappetta D.A., Hocht C., Taira C., Sosnik A. (2010). Efavirenz-loaded polymeric micelles for pediatric anti-HIV pharmacotherapy with significantly higher oral bioavailability. Nanomedicine.

[13] Chiappetta D.A., Hocht C., Taira C., Sosnik A. (2011). Oral pharmacokinetics of the anti-HIV efavirenz encapsulated within polymeric micelles. Biomaterials.

[14] Choi K.C., Bang J.Y., Kim P.I., Kim C., Song C.E. (2008). Amphotericin B-incorporated polymeric micelles composed of poly (D,L-lactide-co-glycolide)/dextran graft copolymer. Int. J. Pharm..

[15] Francis M.F., Lavoie L., Winnik F.M., Leroux J.C. (2003). Solubilization of cyclosporin A in dextran-g-polyethyleneglycolalkyl ether polymeric micelles. Eur. J. Pharm. Biopharm..

[16] Richter A., Olbrich C., Krause M., Kissel T. (2010). Solubilization of Sagopilone, a poorly water-soluble anticancer drug, using polymeric micelles for parenteral delivery. Int. J. Pharm..

[17] Shin H.C., Alani A.W.G., Rao D.A., Rockich N.C., Kwon G.S. (2009). Multi-drug loaded polymeric micelles for simultaneous delivery of poorly soluble anticancer drugs. J. Control Release.

[18] Dolenc A., Kristl J., Baumgartner S., Planinšek O. (2009). Advantages of celecoxib nanosuspension formulation and transformation into tablets. Int. J. Pharm..

[19] Gao L., Liu G.Y., Wang X.Q., Liu F., Xu Y.F., Ma J. (2011). Preparation of a chemically stable quercetin formulation using nanosuspension technology. Int. J. Pharm..

[20] Kayser O., Olbrich C., Yardley V., Kiderlen A.F., Croft S.L. (2003). Formulation of amphotericin B as nanosuspension for oral administration. Int. J. Pharm..

[21] Prabhu S., Ortega M., Ma C. (2005). Novel lipid-based formulations enhancing the *in vitro* dissolution and permeability characteristics of a poorly water-soluble model drug, piroxicam. Int. J. Pharm..

[22] Destache C.J., Belgum T., Christensen K., Shibata A., Sharma A., Dash A.  (2009). Combination antiretroviral drugs in PLGA nanoparticle for HIV-1. BMC Infect. Dis..

[23] Yang J., Grey K., Doney J. (2010). An improved kinetics approach to describe the physical stability of amorphous solid dispersions. Int. J. Pharm..

[24] Jain R.A., Brito L., Straub J.A., Tessier T., Bernstein H. (2008). Effect of powder processing on performance of fenofibrate formulations. Eur. J. Pharm. Biopharm..

[25] Shown I., Banerjee S., Ramchandran A.V., Geckeler K.E., Murthy C.N. (2010). Synthesis of Cyclodextrin and Sugar-Based Oligomers for the Efavirenz Drug Delivery. Macromol. Symp..

[26] Tajber L., Corrigan O.I., Healy A.M. (2005). Physicochemical evaluation of PVP–thiazide diuretic interactions in co-spray-dried composites—Analysis of glass transition composition relationships. Eur. J. Pharm. Sci..

[27] Al-Hamidi H., Edwards A.A., Mohammad M.A., Nokhodchi A. (2010). Glucosamine HCl as a new carrier for improved dissolution behaviour: Effect of grinding. Colloid Surface B.

[28] Barzegar-Jalali M., Valizadeh H., Shadbad M.R.S., Adibkia K., Mohammadi G., Farahani A., Arash Z., Nokhodchi A. (2010). Cogrinding as an approach to enhance dissolution rate of a poorly water-soluble drug (gliclazide). Powder Tech..

[29] Brittain H.G. (2003). X-ray diffraction of pharmaceutical materials. Profiles of Drug Substances, Excipients and Related Methodology.

[30] Gibson M. (2004). Pharmaceutical Preformulation and Formulation—A Practical Guide from Candidate Drug Selection to Commercial Dosage Form.

[31] Otsuka M., Ofusa T., Matsuda Y. (1998). Dissolution improvement of water-insoluble glybuzole by co-grinding and co-melting with surfactants and their physicochemical properties. Colloid Surface B.

[32] Vogt M., Vertzoni M., Kunath K., Reppas C., Dressman J.B. (2008). Cogrinding enhances the oral bioavailability of EMD 57033, a poorly water soluble drug, in dogs. Eur. J. Pharm. Biopharm..

[33] Jagadish B., Yelchuri R., Bindu K., Tangi H., Maroju S., Rao V.U. (2010). Enhanced Dissolution and Bioavailability of Raloxifene Hydrochloride by Co-grinding with Different Superdisintegrants. Chem. Pharm. Bull.

[34] Van den Mooter G., Augustijns P., Blaton N., Kinget R. (1998). Physico-chemical characterization of solid dispersions of temazepam with polyethylene glycol 6000 and PVP K30. Int. J. Pharm..

[35] Bahl D., Bogner R.H. (2008). Amorphization Alone Does Not Account for the Enhancement of Solubility of Drug Co-ground with Silicate: The Case of Indomethacin. AAPS PharmSciTech.

[36] Devilliers M.M. (1996). Influence of agglomeration of cohesive particles on the dissolution behaviour of furosemide powder. Int. J. Pharm..

[37] United States Pharmacopeia 2009. USP’s pending monographs guideline. http//www.usp.org.

[38] Moore J.W., Flanner H.H. (1996). Mathematical comparison of curves with an emphsis on *in vitro* dissolution profiles. Pharm. Technol..

